# SNAT2-mediated regulation of estrogen and progesterone in the proliferation of goat mammary epithelial cells

**DOI:** 10.1007/s00726-024-03382-w

**Published:** 2024-02-23

**Authors:** Tingting Jiang, Xiaoyue Ma, Hanling Liu, Qianqian Jia, Jianguo Chen, Yi Ding, Ming Sun, Hongmei Zhu

**Affiliations:** College of Veterinary Medicine, Huazhong Agricultural University, Wuhan, 430070 China

**Keywords:** SNAT2, Ovarian hormones, Goat mammary epithelial cells, Proliferation, mTOR signaling, Amino acids

## Abstract

The development of the goat mammary gland is mainly under the control of ovarian hormones particularly estrogen and progesterone (P_4_). Amino acids play an essential role in mammary gland development and milk production, and sodium-coupled neutral amino acid transporter 2 (SNAT2) was reported to be expressed in the mammary gland of rats and bovine mammary epithelial cells, which may affect the synthesis of milk proteins or mammary cell proliferation by mediating prolactin, 17β-estradiol (E_2_) or methionine function. However, whether SNAT2 mediates the regulatory effects of E_2_ and P_4_ on the development of the ruminant mammary gland is still unclear. In this study, we show that E_2_ and P_4_ could increase the proliferation of goat mammary epithelial cells (GMECs) and regulate SNAT2 mRNA and protein expression in a dose-dependent manner. Further investigation revealed that SNAT2 is abundantly expressed in the mammary gland during late pregnancy and early lactation, while knockdown and overexpression of SNAT2 in GMECs could inhibit or enhance E_2_- and P_4_-induced cell proliferation as well as mammalian target of rapamycin (mTOR) signaling. We also found that the accelerated proliferation induced by SNAT2 overexpression in GMECs was suppressed by the mTOR signaling pathway inhibitor rapamycin. This indicates that the regulation of GMECs proliferation mediated by SNAT2 in response to E_2_ and P_4_ is dependent on the mTOR signaling pathway. Finally, we found that the total content of the amino acids in GMECs changed after knocking-down and overexpressing SNAT2. In summary, the results demonstrate that the regulatory effects of E_2_ and P_4_ on GMECs proliferation may be mediated by the SNAT2-transported amino acid pathway. These results may offer a novel nutritional target for improving the development of the ruminant mammary gland and milk production.

**Supplementary Information:**

The online version contains supplementary material available at 10.1007/s00726-024-03382-w.

## Introduction

Milk production from goats, sheep and cattle represents an important economic part of the dairy industry. The milk production is directly influenced by factors such as proper mammary gland development, an adequate number of mammary epithelial cells, and sufficient levels of reproductive hormones. Generally, the mammary gland develops through several distinct stages, including the fetus, prepuberty, puberty, pregnancy, lactation and involution. During puberty and pregnancy, the mammary gland grows rapidly under the regulation of reproductive hormones and growth factors to reach a fully developed state and prepare for lactation, while the mammary stroma, such as adipocytes in the fat pad, dedifferentiate, which most likely makes space for the major expansion of the epithelium and provides lipids for milk fat synthesis (Zwick et al. 2018). The development of the mammary gland is mainly regulated by reproductive hormones: estrogen (E) and growth hormone mainly induce the formation of mammary bilayers and promote the extension and branching of mammary ducts (Berryhill et al. 2016); progesterone (P_4_) and prolactin (PRL) play a crucial role in mammary gland alveolar morphogenesis during late pregnancy (Brisken 2002). The ovarian hormones E and P_4_ also interact and strengthen each other synergistically: a previous study reported that human breast tissue receiving E plus P_4_ is more proliferative than tissue receiving E alone, and that a delicate balance of E and P_4_ directs normal breast development (Hofseth et al. 1999).

Amino acids are the basic building blocks of proteins, and constitute almost all cell proteinaceous material including the cytoskeleton, protein components of milk, and signaling molecules (Rezaei et al. 2016). Amino acid transporters, which are mainly distributed over the cell membrane, are critical for cell growth and maintenance since they account for much of the amino acid uptake and transportation by mammalian cells (Palacín et al. 1998). Sodium-coupled neutral amino acid transporter 2 (SNAT2) is a member of the system A family of transporters that primarily mediates the sodium-dependent uptake of small neutral amino acids including alanine and glutamine into cells. The sodium-coupled neutral amino acid transporter 2 (SNAT2) is involved in maintaining the cellular metabolic status by influencing the amino acid content of the cell, which in turn determines the overall size and composition of the intracellular amino acid pool (Jenstad et al. 2009; Franchi-Gazzola et al. 2006). In MCF-7 human breast cancer cells, chronic competitive inhibition of SNAT2 progressively reduced cell proliferation and induced a significant decline in intracellular concentrations of not only SNAT2 AA substrates (e.g., serine, glutamine, alanine, threonine) but of branched chain AAs (leucine, isoleucine and valine). Moreover, the addition of a system A analog elevated mTOR-dependent p70S6K1 phosphorylation, which senses and integrates a variety of environmental cues to regulate organismal growth and homeostasis (Pinilla et al. 2011a). In the rat mammary gland during pregnancy, the mRNA and protein levels of SNAT2 progressively increased. This increase in SNAT2 expression could be regulated by 17-β estradiol (E2) and prolactin (PRL) through enhancing the transcriptional activity of the SNAT2 gene promoter (López et al. 2006; Velázquez-Villegas et al. 2014, 2015). In addition, in bovine mammary epithelial cells, methionine positively regulates milk protein and cell proliferation via the SNAT2-PI3K signaling pathway (Qi et al. 2018). In breast cancer cell lines, SNAT2 was strongly expressed, and SNAT2 knockdown decreased glutamine consumption and cell growth and induced autophagy (Morotti et al. 2021).

Ovarian sex hormones and amino acids are two closely related factors in animal reproduction: rats fed a protein-free diet during early post-implantation stages showed decreased concentrations of essential amino acids in the whole implantation site and maternal liver, and peripheral P_4_ also decreased, but E remained unchanged. However, when rats fed a protein-free diet were given injections of specific dosages of E and P_4_ on days 5–7 of gestation, the dietary protein deficiency-induced decreased concentrations of essential and nonessential amino acids could be restored to control levels (Kohler et al. 1975; Knoll-Köhler et al. 1976). Ovariectomized rats treated with E and P_4_ showed a large increase in the release of glutamate and newly synthesized GABA from preoptic area synaptosomes, which suggests that ovarian steroids may be involved in the control of amino acid neurotransmitter release in the brain area, which is important for female reproductive physiology and behavior (Fleischmann et al. 1990). In our previous research, we confirmed that ovarian hormones regulate tight junctions in GMECs in vitro (Zhu et al. 2022). The central objectives of this study were two-fold: first, to investigate whether ovarian hormones regulate the expression of the amino acid transporter SNAT2 in goat mammary epithelial cells (GMECs) in vitro; and second, to elucidate the role of SNAT2 in mediating the effects of estradiol (E2) and progesterone (P4) on GMECs proliferation and the underlying molecular mechanisms involved.

## Materials and methods

### Animals and tissue collection

Fifteen female hybrids of Boer goats and Macheng black goats at the stage of puberty (approximately 8 months old) were obtained from the Hubei Academy of Agricultural Sciences, Wuhan, Hubei Province, China. The animals were housed and fed in a controlled environment at least 1 week before the experiment. All these goats were in their first parity, and 12 of these goats were optionally collected and mated for pregnancy. The other three goats were assigned to the puberty group. Then, the 12 pregnant goats were optionally assigned to groups of pregnancy day 91 (Pd91), Pd137, lactation day 4 (Ld4), and Ld31, 3 for each group. Mammary tissues were acquired from these four groups of goats using a surgical method under general anesthesia (intravenous injection of sumianxin, 0.01 ml/kg, Veterinary Research Institute, Jilin, China) as previously reported (Zhu et al. 2022). In detail, we first made a skin incision on the avascular area of the mid-abdominal line between two mammary glands, and then, a 1 cm^3^ mammary tissue sample was cut off from an approximately 2–3 cm deep area under the skin of the mammary gland. Finally, we staunched, disinfected and sewed up the wound. The collected mammary tissues were used for cell culture (collected from the three Puberty goats) or were stored in liquid nitrogen immediately for mRNA or protein extraction (collected from all fifteen goats). All animal experiments were approved by the Animal Care and Use Committee of Huazhong Agricultural University (ID number: HZAUGO-2020-001).

### Cell isolation and culture

The cells were isolated from each puberty goat separately. Protocols for GMECs isolation were performed as previously reported (Zhu et al. 2022). In brief, the tissues were first treated with phosphate buffered saline (PBS) and 75% absolute ethanol. Then, after being washed, the tissues were cut into 1 mm^3^ pieces and attached to 60-mm culture dishes with an interval of 1 cm. The dishes were then incubated in a humidified incubator set at 37 °C with 5% CO_2_ in air. Approximately 3–5 h later, DMEM/F12 medium (HyClone, Utah, USA) containing 10% fetal bovine serum (Newzerum, Christchurch, New Zealand) was added to the dishes. About 10 days later, GMECs appeared in the dishes and could be purified from fibroblasts by TE (0.25% trypsin/0.05% EDTA) digestion. 5 × 10^5^ cells/well purified GMECs were seeded in a culture dish and the medium was refreshed every 3 days. After passaging 2–5 times, GMECs were used for subsequent experimental analyses.

### Hormone treatment

For determination of the effect of E_2_ and P_4_ on GMECs, the cells were treated with different concentrations of E_2_ (Sigma, St. Louis, MO, USA) and P_4_ (Sigma). According to previous studies that used concentrations of E_2_ of 10^–9^ M to 10^–8^ M and P_4_ of 10^–6^ M as physiological concentrations to treat goat endometrial epithelial cells or MCF-7 cells in vitro (Yang et al. 2018; Telang 2022), we applied E_2_ and P_4_ concentrations of 10^–8^ M and 10^–6^ M, respectively, as basal concentrations to treat GMECs. In detail, both E_2_ and P_4_ were added to the cultured GMECs. When the concentration of P_4_ was steady at 10^–6^ M (310 ng/ml), the concentrations of E_2_ were divided into 1/16 × 10^–8^ M, 1/8 × 10^–8^ M, 1/4 × 10^–8^ M, 1/2 × 10^–8^ M, 1 × 10^–8^ M, 2 × 10^–8^ M, 4 × 10^–8^ M, 8 × 10^–8^ M, and 16 × 10^–8^ M, and the groups were named 1/16E_2_ + P_4_, 1/8E_2_ + P_4_, 1/4E_2_ + P_4_, 1/2E_2_ + P_4_, E_2_ + P_4_, 2E_2_ + P_4_, 4E_2_ + P_4_, 8E_2_ + P_4_, and 16E_2_ + P_4_. Similarly, when the concentration of E_2_ was steady at 10^–8^ M (2720 pg/ml), the concentrations of P_4_ were 1/16 × 10^–6^ M, 1/8 × 10^–6^ M, 1/4 × 10^–6^ M, 1/2 × 10^–6^ M, 1 × 10^–6^ M, 2 × 10^–6^ M, 4 × 10^–6^ M, 8 × 10^–6^ M, and 16 × 10^–6^ M, and these groups were named E_2_ + 1/16P_4_, E_2_ + 1/8P_4_, E_2_ + 1/4P_4_, E_2_ + 1/2P_4_, E_2_ + P_4_, E_2_ + 2P_4_, E_2_ + 4P_4_, E_2_ + 8P_4_, and E_2_ + 16P_4_. Cells that were not treated with either of these two hormones were designated the control group (concentrations for E_2_ or P_4_ were 0). In the case of rapamycin treatment, 200 mM of rapamycin will be added to the cell culture medium before hormone treatment. All groups of cells were seeded at a confluence of 30% with DMEM/F12 medium containing 10% fetal bovine serum in culture dishes. After 24 h, 48 h, or 72 h, cells incubated with hormone treatment or only with culture medium were used for proliferation tests or were harvested for mRNA and protein extraction. The different treatments of E_2_ + P_4_ were performed on GMECs isolated from at least three different goats at least three times.

### Cell proliferation analysis

For the Cell Counting Kit-8 (CCK-8) assay, GMECs (4000 cells per well) were seeded in 96-well plates with different concentrations of hormone treatment for the proliferation assay. After 24 h, 48 h, or 72 h, ten microliters of CCK-8 reagent (Biosharp, Wuhan, China) was added to each well of the plate and then incubated at 37 °C for 2 h. Each hormone treatment of GMECs was performed with six replicates. The absorbance of each well was obtained using a microplate reader (BMG LABTECH, Offenburg, Baden-Wurttemberg, Germany) at a wavelength of 450 nm.

For 5-ethynyl-20-deoxyuridine (EdU) incorporation assay, GMECs were seeded in 24-well plates at a confluence of 30%. After 24 h of hormone treatment, ten micromoles of EdU reagent was added to each well of the plate, and then, the plate was incubated at 37 °C for 2 h. The experiment was then carried out according to the instructions from the manufacturer of the BeyoClick™ EdU Cell Proliferation Kit with Alexa Fluor 555 (C0075S, Beyotime, Shanghai, China). Each hormone treatment of GMECs was performed with three replicates. Finally, the cells were incubated with Hoechst 33342 for 10 min in the dark for nuclear staining. The results were finally visualized under a microscope. The number of proliferating cells was quantified using Image J software (version 1.53t, National Institute of Health, Bethesda, MD, USA).

### Real-time quantitative PCR

Total RNA of GMECs was isolated using AG RNAex Pro Reagent (Accurate Biology, Hunan, China). Then, 1 µg of total RNA from each sample was reverse transcribed into cDNA using a Thermo First cDNA Synthesis Kit (Hlingene Corporation, Shanghai, China). Quantitative real-time PCR (qRT-PCR) was performed according to the protocol of the RealTime FAST SYBR mix (Hlingene Corporation). The specific primers for detecting the SNAT2 (XM_018047891.1) transcript were as follows: forward primer 5′-GCAAGCAGGAACGATGAG-3′ and reverse primer 5′-CCATGCCAATGTTGTCTCTT-3′. The β-actin mRNA was used as an internal control, and the primers targeting β-actin (NM_001009784.3) were as follows: forward primer 5′-GGATGATGATATTGCTGCGCTC-3′ and reverse primer 5′-TCTCCATGTCGTCCCAGTTGGT-3′. These reactions were repeated three times for each sample. The relative expression of SNAT2 mRNA was calculated using the 2^−△△Ct^ method, where ΔΔCt = ΔCt1(experimental group) − ΔCt2(control group) and ΔCt = Ct_targetgene_ − Ct_β-actin_.

### Western blotting

After treatment with varying concentrations of hormones, the GMECs were washed three times with PBS for 30 s each, then lysed with cold RIPA lysis buffer (20 μl/mg, Beyotime, Shanghai, China) containing 1% phenylmethanesulfonyl fluoride (PMSF) protease inhibitor to collect total protein. The extracted protein in each sample was boiled with loading buffer at 100 °C for 10 min. Then, these protein samples were separated using 12% SDS-PAGE gels and transferred to polyvinylidene fluoride membranes (Biosharp, Wuhan, China). The Multicolor Prestained Protein Ladder (WJ102, Shanghai Epizyme Biomedical Technology Co., Ltd, Shanghai, China) was used as a molecular weight marker. After blocking with 5% skim milk powder for 2 h, the membranes were incubated overnight at 4 °C with the following primary antibodies: anti-SLC38A2 antibody (1:1000, Bioss, Beijing, China), rabbit anti-mTOR polyclonal antibody (1:1000, A11355, ABclone, Wuhan, China), phospho-mTOR (S2448) antibody (1:500, CY6571, Abways, Beijing, China), eIF4EBP1 rabbit pAb (1:1000, A1248, ABclone, Wuhan, China), phospho-eIF4EBP1-T37/46 rabbit pAb (1:500, ABclone, Wuhan, China), p70S6 Kinase 1 (S6K1) rabbit pAb (1:1000, A16968, ABclone, Wuhan, China), phospho-S6K1 (T421 + S424) antibody (1:500, CY5261, Abways, Beijing, China), and β-actin mouse mAb (1:4000, AC004, ABclone, Wuhan, China). After being washed in Tris-buffered saline and Tween 20 (TBST) 5 times, the membranes were incubated with the corresponding horseradish peroxidase (HRP)-conjugated secondary donkey anti-rabbit IgG (1:4000, ABclone, Wuhan, China) or secondary donkey anti-mouse IgG (1:4000, ABclone, Wuhan, China) at room temperature for 2 h. These experiments were performed on each sample with at least three replicates for each protein. The target proteins were finally visualized with chemiluminescence ECL (Biosharp, Wuhan, China). The protein levels normalized to the β-actin were quantified with Image-Pro plus 6.0 software (Media Cybernetics, Inc., Silver Spring, MD, United States). The objective bands used for analysis can be viewed in the supplementary material.

### SNAT2 overexpression

Goat genomic DNA was extracted from the mammary gland and used to amplify SNAT2 (XM_018047891.1). The primers for targeting SNAT2 DNA sequences were as follows: forward primer, 5′-CCCAAGCTTCCATGAAGAAAGCTGAAATGGGAAGG-3′ (Hind-III) and reverse primer, 5′-CCGGAATTCACACTGGCGTCAAATGGACT-3′ (EcoR-I). The amplified SNAT2 fragment was subcloned into an expression vector (pEGFP-C1) and completely sequenced with a sequencing company (Genecreate, Wuhan, China). The recombinant plasmid was designated pEGFP-SNAT2-C1 (Supplementary Fig. 1). The Overexpression (OE) group of GMECs was transfected with the pEGFP-SNAT2-C1 plasmid using Lipofectamine 2000 (11668027, Invitrogen, Carlsbad, CA). Cells transfected with the empty vector (pEGFP-C1) were used as negative controls (EV group). Cells were harvested for mRNA and protein assays 72 h post-transfection.

### SNAT2 knockdown

Three specific siRNAs against different sequences of SNAT2 mRNA, and the negative control siRNA were synthesized by GenePharma (Shanghai, China). The most effective siRNA from these three siRNAs was selected by transfection into GMECs using Lipofectamine 2000 (Invitrogen) (Supplementary Fig. 2). The most effective siRNA sequences against SNAT2 were as follows: sense, 5′-GCUCUGUUCUUCCUGCUAATT-3′ and antisense, 5′-UUAGCAGGAAGAACAGAGCTT-3′. Cells transfected with this siRNA were designated siSNAT2 group. Cells transfected with negative control siRNA were used as a negative control (NC group). Twenty-four hours after siRNA transfection, GMECs were harvested to assess cell proliferation and mRNA or protein expression.

### Amino acids detection

The intracellular amino acid content of the GMECs treated with 4E_2_ + P_4_ (4E_2_/P_4_ group), negative siRNA and 4E_2_ + P_4_ (NC + 4E_2_/P_4_ group), SNAT2 siRNA and 4E_2_ + P_4_ (siRNA + 4E_2_/P_4_ group), EV vector and 4E_2_ + P_4_ (EV + 4E_2_/P_4_ group), and SNAT2 overexpression vector and 4E_2_ + P_4_ (OE + 4E_2_/P_4_) were tested using an Amino Acid Content Assay Kit (BC1570, Solarbio, Beijing, China). The principle for this kit is that the α-amino group of the amino acids can react with ninhydrin hydrate to produce a blue-purple compound with a characteristic absorption peak at 570 nm; the amino acid content can be calculated by measuring the absorbance at 570 nm.

### Statistical analysis

Data were analyzed using IBM SPSS Statistics 17.0 software (IBM, Armonk, New York, NY, USA). Prior to one-way ANOVA analysis, Levene's test was used to assess the homogeneity of variances between groups, showing no significant difference (*P* > 0.05). The Kolmogorov–Smirnov test showed that the samples followed normal distribution (*P* > 0.05). Differences in cell viability, EdU-positive cells, SNAT2 mRNA and protein expression, and intracellular amino acid levels between groups of hormone-treated GMECs or different stages of the mammary gland were analyzed by one-way ANOVA. Differences in SNAT2, mTOR, p-mTOR, S6K1, p-S6K1, 4EBP1, and p-4EBP1 mRNA or protein expression between groups of GMECs with SNAT2 interference or overexpression were analyzed by Student’s t test. All the data generated were normally distributed and have not been transformed in some fashion prior to statistics. All data are presented as the means ± SD. Statistical significance was defined at values of *P* < 0.05, *P* < 0.01 or *P* < 0.001. The statistical significance was indicated by different alphabetical letters or asterisks.

## Results

### The effect of different concentrations of E_2_ and P_4_ on the proliferation of GMECs

**Fig. 1:**
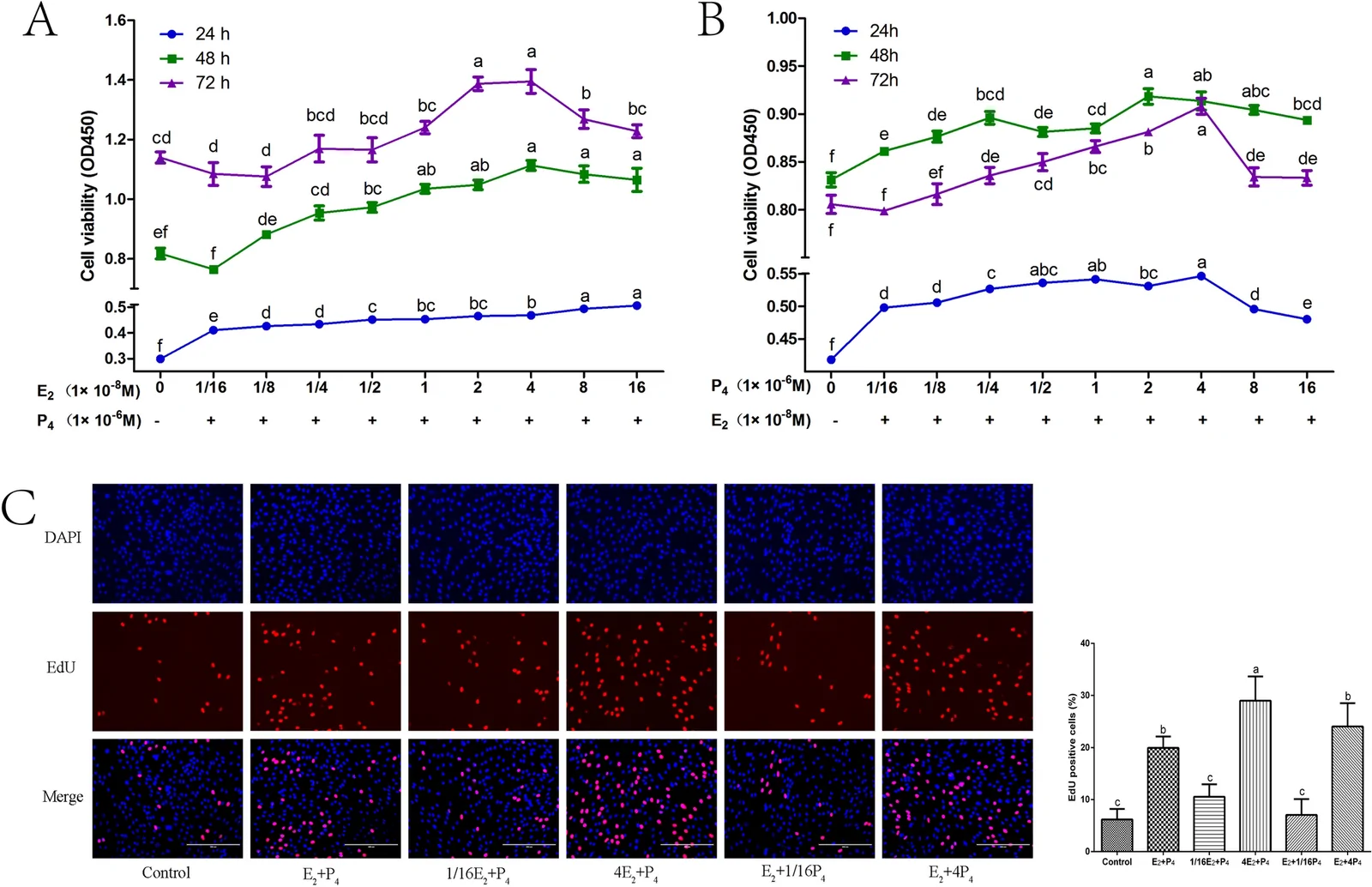
Regulation of E_2_ and P_4_ on proliferation of GMECs. **A** The GMECs were treated with different concentrations of E_2_ plus a specific concentration of P_4_ (0, 1/16E_2_ + P_4_, 1/8E_2_ + P_4_, 1/4E_2_ + P_4_, 1/2E_2_ + P_4_, E_2_ + P_4_, 2E_2_ + P_4_, 4E_2_ + P_4_, 8E_2_ + P_4_, 16E_2_ + P_4_) for 24, 48 and 72 h. The cell viability was determined using the CCK-8 assay. **B** The GMECs were treated with different concentrations of P_4_ plus a specific concentration of E_2_ (0, E_2_ + 1/16P_4_, E_2_ + 1/8P_4_, E_2_ + 1/4P_4_, E_2_ + 1/2P_4_, E_2_ + P_4_, E_2_ + 2P_4_, E_2_ + 4P_4_, E_2_ + 8P_4_, E_2_ + 16P_4_) for 24, 48 and 72 h. The cell viability was determined using the CCK-8 assay. **C** The GMECs were treated with different concentrations of E_2_ and P_4_ (0, E_2_ + P_4_, 1/16E_2_ + P_4_, 4E_2_ + P_4_, E_2_ + 1/16P_4_, E_2_ + 4P_4_) for 24 h, and the proliferation of GMECs was examined by EdU. Scale bar represents 200 μm. Values are means ± SD form at least three independent experiments. Values with different lowercase letter indicate significant difference (*P* < 0.05). GMECs, goat mammary epithelial cells; CCK-8, cell counting kit-8; EdU, Ethynyl-20 –deoxyuridine

GMECs were treated with different concentrations of E_2_ and P_4_ as shown in Fig. 1A and B. The CCK-8 assay showed that the proliferation of GMECs was gradually elevated when E_2_ concentrations increased from 1/16E_2_ to 16E_2_ 24 h after hormone treatment in cells, whereas 48 h and 72 h later, the proliferation of GMECs reached its peak when treated with 4E_2_ + P_4_ (Fig. 1A). When the P_4_ concentration changed, the proliferation of GMECs reached its peak at 24 h, 48 h, or 72 h after 4P_4_ + E_2_ treatment (Fig. 1B). To further verify the effect of E_2_ and P_4_ on the proliferation of GMECs, we used an EdU assay to detect the proliferation of GMECs treated with E_2_ + P_4_, 1/16E_2_ + P_4_, 4E_2_ + P_4_, E_2_ + 1/16P_4_, and E_2_ + 4P_4_. The results showed that the EdU-positive cells in the 4E_2_ + P_4_ group and E_2_ + 4P_4_ group were significantly more abundant than those in the 1/16E_2_ + P_4_ group (4E_2_ + P_4_, 29 ± 5% vs. 1/16E_2_ + P_4_, 10.53 ± 2.4%, *P* < 0.001) and E_2_ + 1/16P_4_ group (E_2_ + 4P_4_, 24.04 ± 4.49% vs. E_2_ + 1/16P_4_, 7.04 ± 3.04%, *P* < 0.001), after 24 h of hormone treatment (Fig. 1C). These results suggested that E_2_ and P_4_ played positive roles in accelerating the proliferation of GMECs in a dose- and time-dependent manner.

### The effect of E_2_ and P_4_ on SNAT2 mRNA and protein expression

**Fig. 2:**
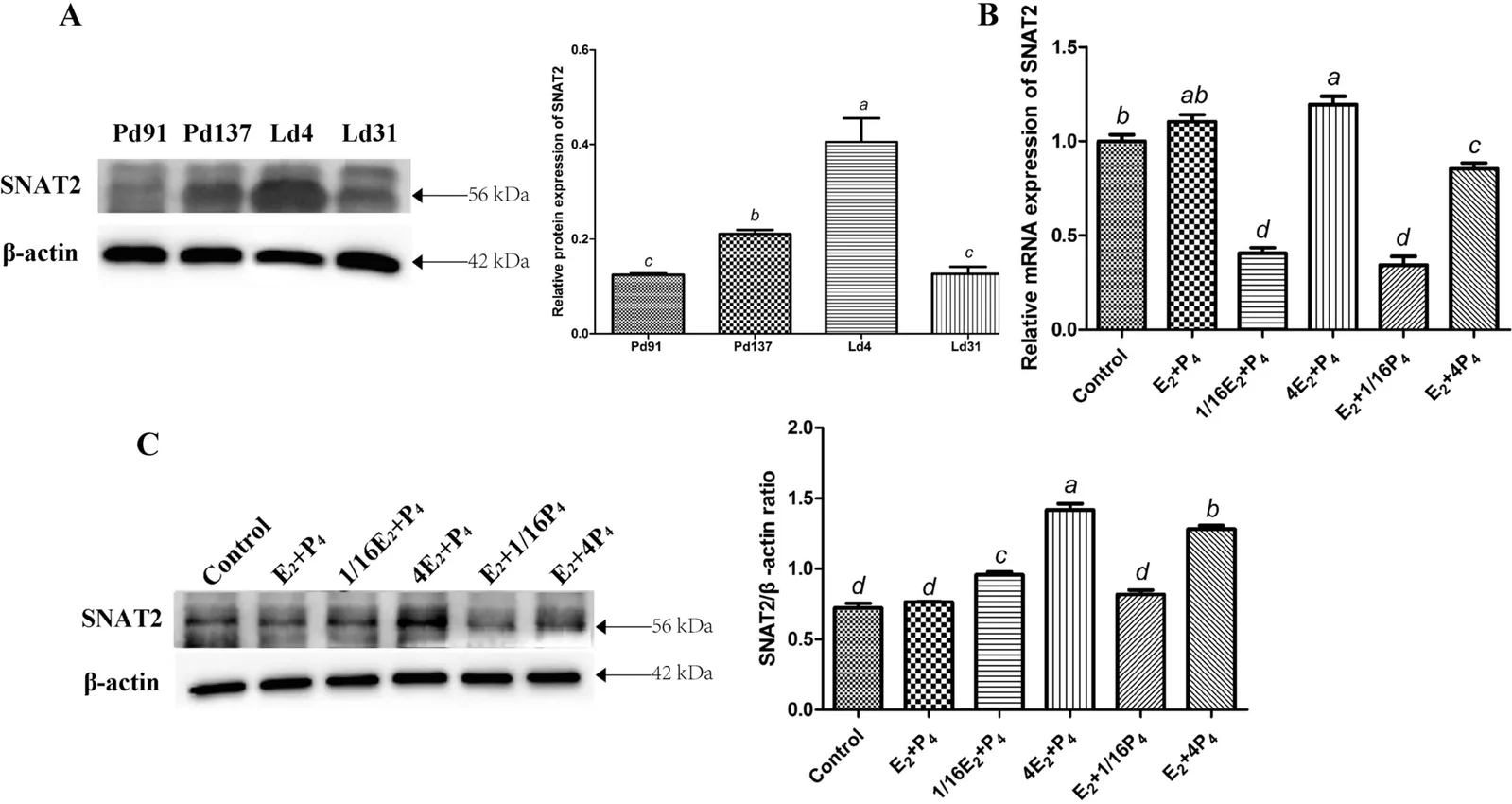
Regulation of E_2_ and P_4_ on SNAT2 mRNA and protein expression. **A** The mammary SNAT2 protein expression of goats at stages of Pd91, Pd137, Ld4 and Ld31 was detected by western blot analysis. **B** The mRNA expression of SNAT2 in GMECs under the treatment of different concentrations of E_2_ and P_4_ was detected by real-time PCR. **C** The protein expression of SNAT2 in GMECs under the treatment of different concentrations of E_2_ and P_4_ was detected by western blot analysis. Data were the means ± SD from at least three independent experiments. Values with different lowercase letter indicate significant difference (*P* < 0.05). Pd91, pregnancy day 91; Pd137, pregnancy day 137; Ld4, lactation day 4; Ld31, lactation day 31

SNAT2 protein expression in the goat mammary gland during different stages of pregnancy and lactation was assayed with Western blot analysis. The results showed that during pregnancy, the mammary protein levels of SNAT2 on Pd137 were higher than those on Pd91 (*P* < 0.001), whereas during lactation, the mammary SNAT2 protein levels on Ld4 were higher than those on Ld31 (*P* < 0.001) (Fig. 2A). These results suggested that SNAT2 expression may be related to mammary gland development and lactation.

To further explore whether SNAT2 mediated the effects of E_2_ and P_4_ on the proliferation of GMECs, we used concentrations of E_2_ + P_4_, 1/16E_2_ + P_4_, 4E_2_ + P_4_, E_2_ + 1/16P_4_, and E_2_ + 4P_4_ to treat GMECs to assess SNAT2 mRNA and protein expression. The results demonstrated that the mRNA levels of SNAT2 increased whenever E_2_ concentrations increased from 1/16E_2_ to 4E_2_ (*P* < 0.001) or P_4_ concentrations increased from 1/16P_4_ to 4P_4_ (*P* < 0.001) (Fig. 2B). Similarly, compared to the control group without hormonal treatment, SNAT2 protein levels were greatly enhanced whenever E_2_ concentrations increased from 1/16E_2_ to 4E_2_ (*P* < 0.001) or P_4_ concentrations increased from 1/16P_4_ to 4P_4_ (*P* < 0.001) (Fig. 2C). These results suggest that the trends of SNAT2 mRNA and protein expression are similar to those of GMECs proliferation under the treatment of specific concentrations of E_2_ and P_4_. Thus, we speculate that SNAT2 may mediate the regulatory effects of E_2_ and P_4_ on GMECs proliferation.

### SNAT2-mediated regulation the proliferation of GMECs under E_2_ and P_4_ treatment

**Fig. 3:**
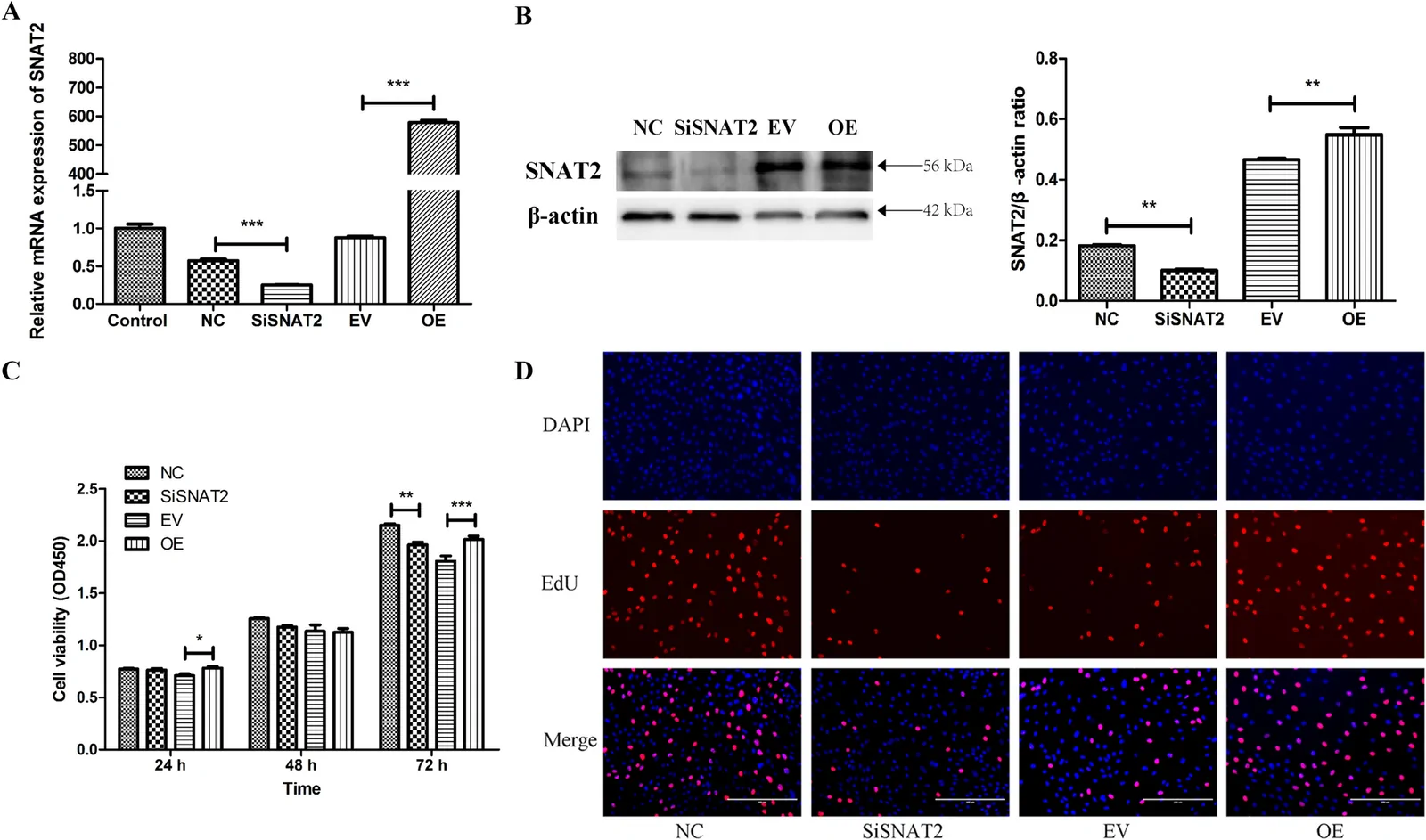
The role of SNAT2 in the regulation of E_2_ and P_4_ on proliferation of GMECs. **A** SNAT2 mRNA and **B** protein expression after being interfered by siRNA and being overexpressed. **C** CCK-8 assay on the cell viability of GMECs after SNAT2 was being interfered or overexpressed. **D** EdU assay on the proliferation of GMECs after SNAT2 was being interfered or overexpressed. Values are means ± SD from at least three independent experiments. ***P < 0.001, **P < 0.01, *P < 0.05, *compared with EV or NC group. NC, negative control; SiSNAT2, SNAT2 siRNA; EV, empty vector; OE, SNAT2 overexpression vector

To verify whether SNAT2 mediates the regulatory effects of E_2_ and P_4_ on GMEC proliferation, we knocked down and overexpressed SNAT2 by transfecting GMECs with siRNAs against SNAT2 mRNA or an overexpression vector (Fig. 3A and B). Then, the proliferation of SNAT2-knocked down and SNAT2-overexpressing GMECs was detected with CCK-8 and EdU assays after 24 h of E_2_ and P_4_ treatment. The results showed that SNAT2 interference inhibited cell viability (SiSNAT2 vs. NC, *P* = 0.005), whereas SNAT2 overexpression enhanced cell viability (OE vs. EV, *P* = 0.001) (Fig. 3C). EdU proliferation assays further demonstrated that EdU-positive cells were reduced (SiSNAT2 vs. NC, *P* = 0.007) or increased (OE vs. EV, *P* < 0.001) compared with those in the negative control group after SNAT2 was knocked down or overexpressed in GMECs (Fig. 3D). These results revealed that SNAT2 is involved in regulating the proliferation of GMECs under stimulation E_2_ and P_4_.

### SNAT2-mediated regulation the proliferation of GMECs through mTOR signaling under E_2_ and P_4_ treatment

**Fig. 4:**
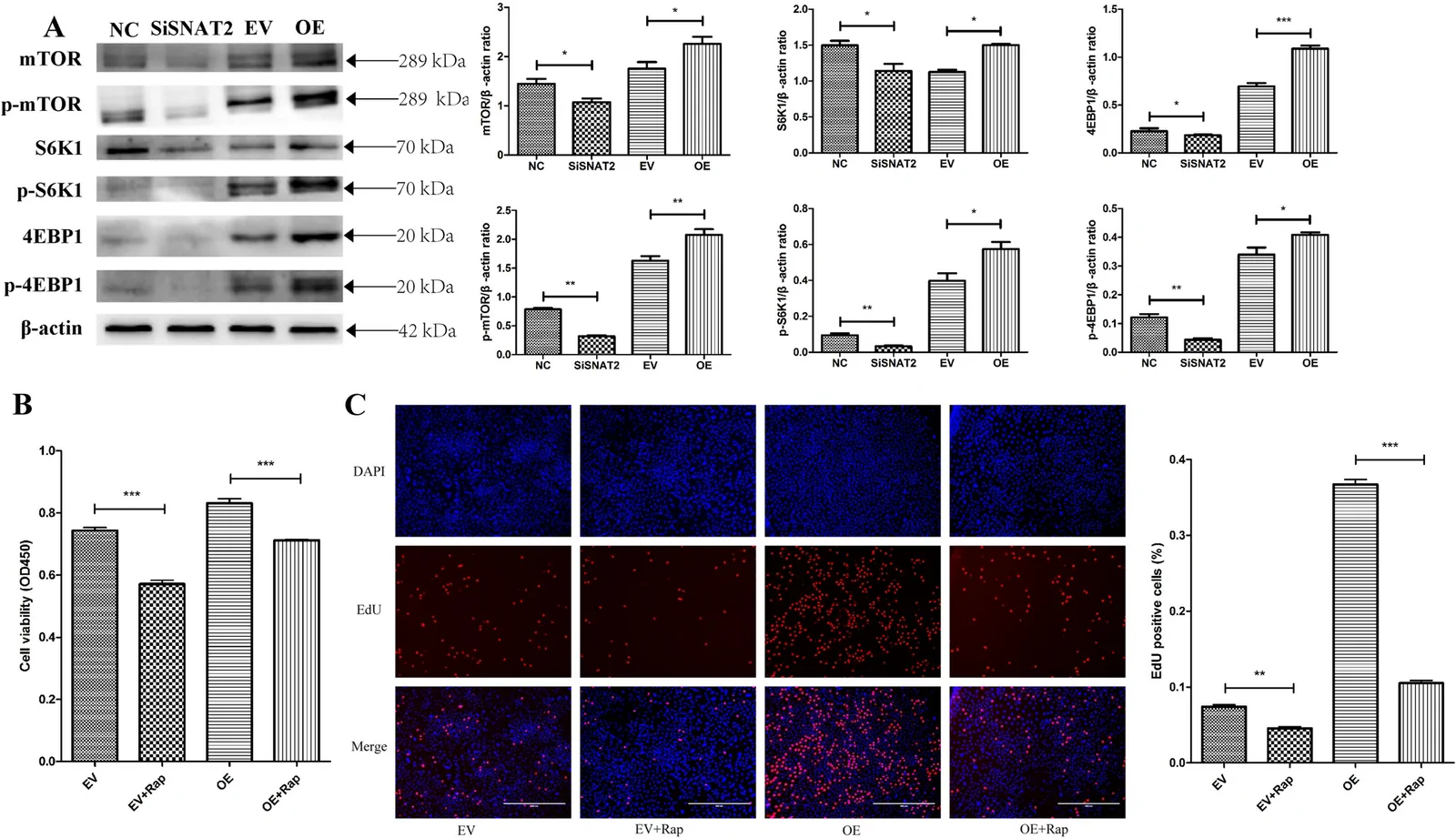
The role of mTOR signaling on SNAT2-mediated regulation of E_2_ and P_4_ on proliferation of GMECs. **A** Western blot analysis on the protein expression of mTOR, p-mTOR, S6K1, p-S6K1, 4EBP1, p-4EBP1 after SNAT2 was interfered and overexpressed in GMECs. **B** CCK-8 assay on the cell viability of GMECs after SNAT2 was overexpressed and treated with Rapamycin. **C** The proliferation of GMECs overexpressed with SNAT2 and treated with Rapamycin was examined by EdU. Data were shown as means ± SD from at least three independent experiments. Values with different lowercase letter indicate significant difference (*P* < 0.05). Rap, Rapamycin

Activation of the mTOR signaling pathway regulates the proliferation of a variety of mammalian cells. Previous studies have demonstrated that the inhibition of mammalian target of rapamycin (mTOR) reduces the proliferation of bovine mammary epithelial cells (Li et al. 2020). To explore whether mTOR signaling participates in the effect of SNAT2 on E_2_- and P_4_-induced GMEC proliferation, we determined the expression of mTOR signaling pathways in GMECs treated with E_2_ and P_4_ after SNAT2 was overexpressed or knocked down. The results demonstrated that SNAT2 overexpression significantly increased the total protein levels of mTOR (*P* = 0.026), S6K1 (*P* = 0.035), and 4EBP1 (*P* < 0.001) as well as the phosphorylation of mTOR (*P* = 0.003), S6K1 (*P* = 0.045) and 4EBP1 (*P* = 0.023), whereas SNAT2 knockdown inhibited the expression of these proteins (*P* < 0.05) (Fig. 4A). These results reveal that SNAT2 may be a positive regulator of the mTOR/S6K1/4EBP1 signaling pathway during the proliferation of GMECs.

To further verify whether mTOR signaling participates in SNAT2-mediated regulation of E_2_ and P_4_ in GMEC proliferation, we treated GMECs 200 nM rapamycin (an inhibitor of mTOR, the optimal concentration was selected in pretest, see Supplementary Fig. 3) after SNAT2 was overexpressed. The CCK-8 and EdU assays showed that SNAT2 overexpression promoted the proliferation of GMECs (CCK-8, OE + rap vs. OE, *P* < 0.001; EdU, OE + rap vs. OE, *P* < 0.001) while rapamycin treatment inhibited SNAT2-induced cell proliferation (Fig. 4B, C). These results showed that SNAT2 may promote E_2_- and P_4_-induced cell proliferation through mTOR signaling.

### The regulatory effects of E_2_ and P_4_ on GMECs proliferation may be mediated by SNAT2-transported amino acids

**Fig. 5:**
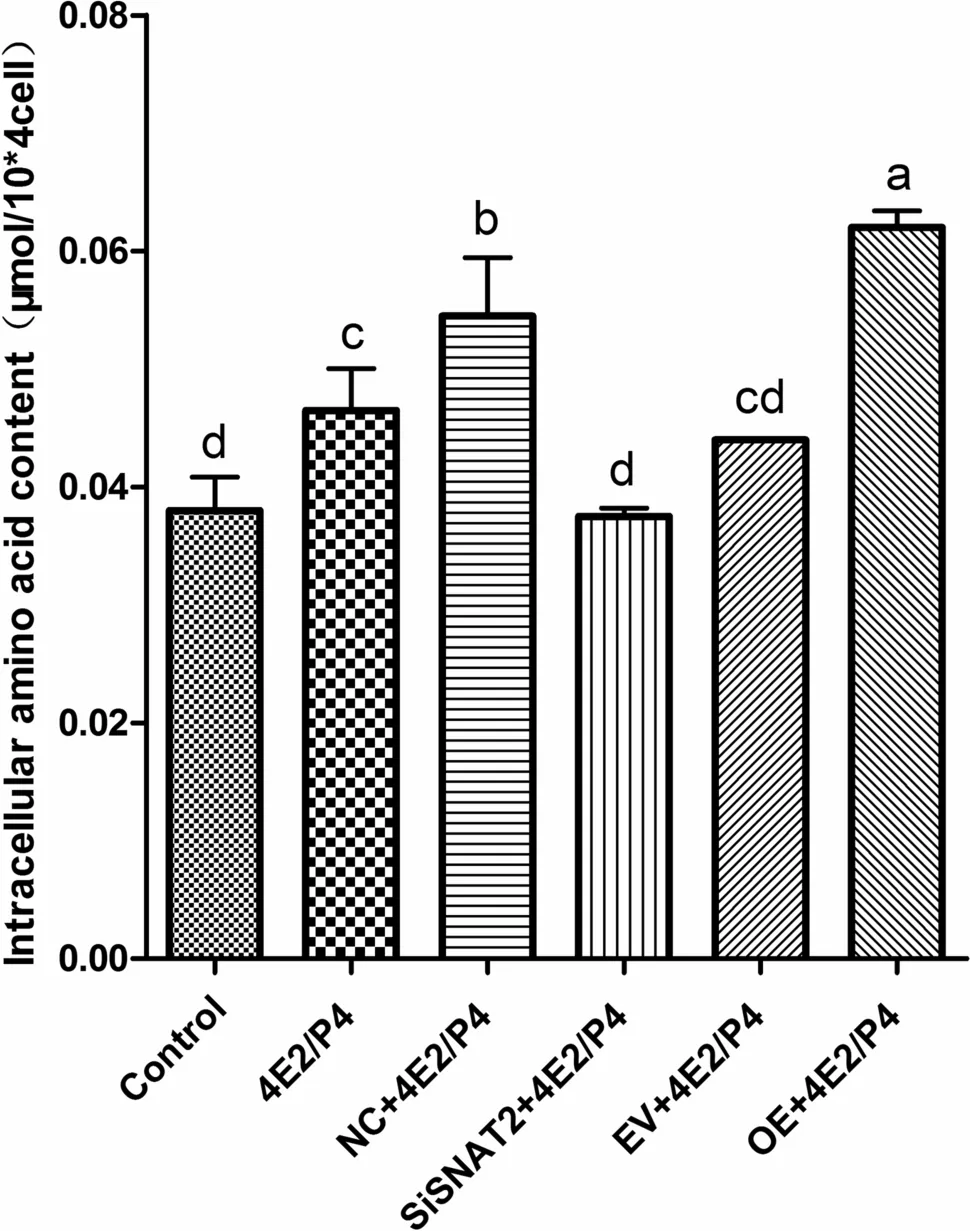
The assessment of intracellular amino acid level in SNAT2 knocking-down and overexpressing GMECs treated with ovarian hormones. Data were shown as means ± SD from at least three independent experiments. Values with different lowercase letter indicate significant difference (*P* < 0.05)

To clarify whether SNAT2 interference and overexpression affect cell amino acid levels, we assessed the intracellular amino acid level in SNAT2-knockdown and -overexpressing GMECs treated with ovarian hormones. As shown in Fig. 5, the results demonstrated that GMECs treated with E_2_ and P_4_ had elevated intracellular amino acid levels compared with those of the control group (4E_2_/P_4_ vs. control, *P* = 0.023). This result suggests that ovarian hormones may promote cell proliferation via amino acids. However, the amino acid level in the siRNA + 4 E_2_/P_4_ group was reduced to control levels compared with that in the NC + 4E_2_/P_4_ group (*P* < 0.001); in contrast, the amino acid level in the OE + 4 E_2_/P_4_ group was increased compared with that in the EV + 4 E_2_/P_4_ group (*P* < 0.001), demonstrating that SNAT2 interference and overexpression inhibit or accelerate the intracellular amino acid content. These results indicate that ovarian hormones may regulate cell proliferation through SNAT2-transported amino acids.

## Discussion

The development and homeostasis of the mammary gland are highly dependent upon the actions of ovarian hormones. During pregnancy, the mammary gland undergoes ductal development and alveolar structure establishment, which are prepared for the onset of lactogenesis. Ductal elongation is mainly directed by estradiol (E2), while ductal branching and alveolar budding are mainly influenced by progesterone (P4). During pregnancy in goats, the plasma estradiol (E_2_) concentration remained at basal levels (3 pg/ml) in the first month of gestation. It then gradually increased from 312 pg/ml in the second month of gestation. Finally, E_2_ reached maximal levels (~ 22 pg/ml) approximately 1 month prior to parturition. The plasma P_4_ concentrations increased abruptly by the first week of gestation (2–9 ng/ml) and remained at high levels from the second month to the early stage of the fifth month of gestation in vivo (6–16 ng/ml) (Kandiel et al. 2010; Yazici et al. 2018). However, in the current study of GMECs in vitro, we arranged the concentrations of E_2_ from 1/16E_2_ (170 pg/ml) to 16E_2_ (43,520 pg/ml) and the concentrations of P_4_ from 1/16P_4_ (19.375 ng/ml) to 16P_4_ (4960 ng/ml); the concentrations were referenced from previous studies and may be beneficial to study the effects of hormones on the proliferation of goat mammary epithelial cells in vitro (Yang et al. 2018; Telang 2022; Zhu et al. 2022). The current results demonstrated that administration of E_2_ and P_4_ both facilitated proliferation of GMECs in a dose-dependent manner and that this effect was most obvious when the E_2_ concentration increased to 10,880 pg/ml (4E_2_) and when the P_4_ concentration increased to 1240 ng/ml (4P_4_) after 24 or 48 h of treatment. These results demonstrated that E_2_ exerts its effect on the development of the goat mammary gland, which may mainly occur at mid-pregnancy from 2 to 5 months. Regarding P_4_, the minimum concentration 1/16 P_4_ (~ 19.38 pg_/_ml) in the current study promoted cell viability 24 h after treatment, suggesting that P_4_ may affect the development of the mammary gland throughout the whole pregnancy.

Cell growth and proliferation depend on an adequate supply of nutrients (glucose, amino acids, fatty acids, etc.) to support the biosynthesis of a variety of macromolecules (including proteins, nucleic acids, and complex lipids), as well as the energy requirements associated with growth and renewal processes. The amino acid transporter SNAT2, which is regulated by amino acid deprivation (Morotti et al. 2021), pharmacological stresses, and hormonal signals (such as E_2_ and PRL) (Velázquez-Villegas et al. 2014, 2015), is widely expressed in mammalian tissues such as the brain, skeletal muscle(Mazzulla et al. 2021), liver, placenta (Vaughan et al. 2021), and mammary gland (López et al. 2006). Previous studies reported that SNAT2 protein was abundant in the rat mammary gland or mammary gland explants during pregnancy and lactation, and that SNAT2-PI3K signaling mediated the effect of methionine on bovine mammary epithelial cell proliferation (Qi et al. 2018). Consistent with previous reports, the current study found that SNAT2 protein expression was enhanced in late pregnancy (Pd137) and early lactation (Ld4) in comparison with that in mid-pregnancy (Pd91) or late lactation (Ld31). These results suggested the important role of SNAT2 in mammary gland development and lactation. In addition, the similar expression pattern of SNAT2 with the proliferation of GMECs under the treatment of specific concentrations of E_2_ or P_4_ further demonstrated that SNAT2 may mediate the effect of ovarian hormones on the proliferation of GMECs and that not only E_2_ but also P_4_ regulates the mRNA and protein expression of SNAT2. Moreover, since SNAT2 is an amino acid transporter, Since SNAT2 is an amino acid transporter, it is possible that SNAT2 participates in the regulation of GMECs proliferation through the transport of amino acids under the influence of estradiol and progesterone. From the current study, we also speculate that the higher estrogen and progesterone levels in late pregnancy may promote the expression of SNAT2 proteins, which facilitate the proliferation of GMECs. During early lactation on Ld4, the concentrations of E_2_ and P_4_ decreased to basal levels (Kandiel et al. 2012), and the SNAT2 abundance during this stage may suggest that there are other factors (such as PRL) that regulate the expression of SNAT2 since the PRL levels are higher in the first week than in the eighth week after parturition in goats (Hashizume et al. 1999). In addition, the higher expression of SNAT2 in early lactation on Ld4 than that on Ld31 may also be the result of the need for much more amino acid and milk protein synthesis for colostrum on Ld4 (Palii et al. 2004). These results suggest that SNAT2 may play a role in milk synthesis in response to PRL and amino acid levels in goats, which needs further investigation.

The mammalian target of rapamycin (mTOR) is a serine/threonine protein kinase and interacts with several proteins to form two distinct complexes named mTOR complex1 (mTORC1) and 2 (mTORC2). In response to growth factors, energy levels, and amino acids, mTORC1 is involved in gene transcription, protein translation initiation, cell growth, and apoptosis (Sancak et al. 2010), can phosphorylate its downstream signaling molecules S6 kinase 1 (70S6K1) and eukaryotic translation initiation factor 4E (eIF4E)-binding protein 1(4EBP1). Studies have reported that amino acids can directly target mTORC1 through the Ragulator-Rag complex, and then, mTORC1 is activated to affect cell proliferation. A reduction in amino acid transporter SNAT2 activity in L6 myotubes is associated with impaired mTORC1 activation, and complete inhibition of SNAT2 with 10 mM α-methylaminoisobutyric acid (MeAIB) in myoblasts impaired protein synthesis, stimulated protein degradation, and decreased total protein without loss of cell viability (Evans et al. 2007). Consistent with a previous report (Pinilla et al. 2011b), the current research showed that SNAT2 overexpression in GMECs significantly enhanced the cell protein expression of p-mTOR, p-4EBP1, and p-S6K1, as well as the proliferation of GMECs and vice versa. Furthermore, mTOR inhibition with rapamycin in GMECs inhibited GMEC proliferation which was induced by SNAT2 overexpression. These results demonstrated that the role of SNAT2 in participating E_2_ and P_4_ in regulating GMECs proliferation is activated through mTOR/4EBP1/S6K1 signaling pathways. In addition, similar variations in the total protein levels of mTOR, S6K1, and 4EBP1 suggest that SNAT2 regulates both the phosphorylation of mTOR, S6K1 and 4EBP1 and the total protein metabolism of these molecules in GMECs.

## Conclusion

**Fig. 6:**
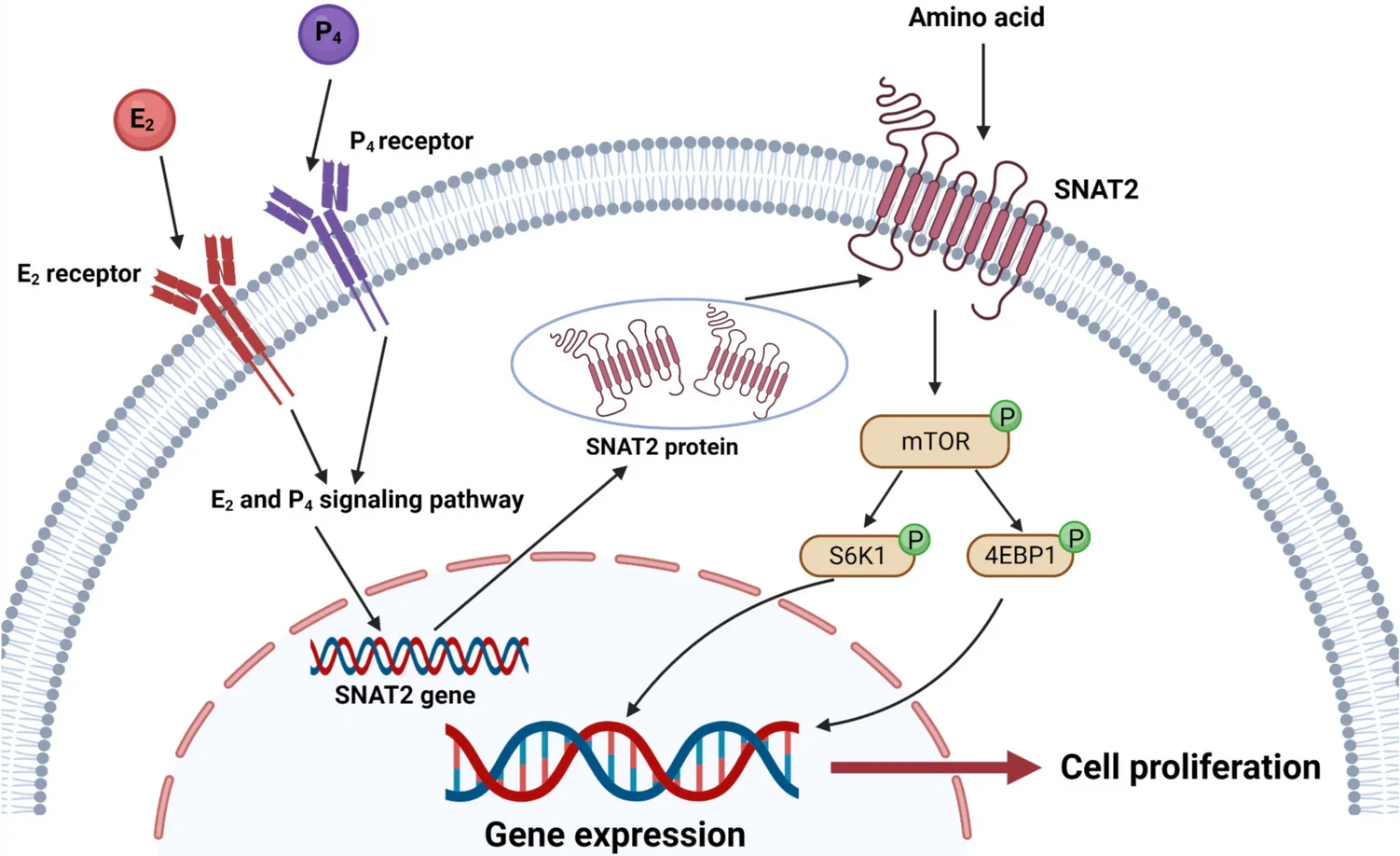
Scheme summarizing the effect and mechanism of SNAT2 mediating the effect of E_2_ and P_4_ on proliferation of GMECs. E_2_ and P_4_ may regulate the SNAT2 mRNA and protein expression, and then transmembrane SNAT2 activates the mTOR, S6K1, and 4EBP1 which promote the proliferation of GMECs. Created with BioRender.com

In summary, SNAT2 knockdown or overexpression in GMECs indeed affected the intracellular amino acid levels (Fig. 5). This finding indicates that SNAT2 mediates the effects of E_2_ and P_4_ on GMECs proliferation not only by activating the mTOR/4EBP1/S6K1 signaling pathways but also by transporting amino acids into cells (Fig. 6). The current research provides a theoretical basis for further study on the mechanism of mammary gland development and postpartum hypogalactia in response to hormones in ruminant animals. However, whether SNAT2 is involved in milk lactation and the underlying mechanisms require further investigation.

## Supplementary Information

Below is the link to the electronic supplementary material.

Supplementary file1 (DOCX 10106 KB)

## Data Availability

The data underlying this article will be shared on reasonable request to the corresponding author.

## References

[CR1] Berryhill GE, Trott JF, Hovey RC (2016) Mammary gland development–It’s not just about estrogen. J Dairy Sci 99(1):875–883 26506542 10.3168/jds.2015-10105 PMC5068565

[CR2] Brisken C (2002) Hormonal control of alveolar development and its implications for breast carcinogenesis. J Mammary Gland Biol Neoplasia 7(1):39–48 12160085 10.1023/a:1015718406329

[CR3] Evans K, Nasim Z, Brown J, Butler H, Kauser S, Varoqui H, Erickson JD, Herbert TP, Bevington A (2007) Acidosis-sensing glutamine pump SNAT2 determines amino acid levels and mammalian target of rapamycin signalling to protein synthesis in L6 muscle cells. J Am Soc Nephrol 18(5):1426–1436 17429052 10.1681/ASN.2006091014

[CR4] Fleischmann A, Makman MH, Etgen AM (1990) Ovarian steroids increase veratridine-induced release of amino acid neurotransmitters in preoptic area synaptosomes. Brain Res 507(1):161–163. 10.1016/0006-8993(90)90538-m 1967974 10.1016/0006-8993(90)90538-m

[CR5] Franchi-Gazzola R, Dall’Asta V, Sala R, Visigalli R, Bevilacqua E, Gaccioli F, Gazzola GC, Bussolati O (2006) The role of the neutral amino acid transporter SNAT2 in cell volume regulation. Acta Physiol 187(1–2):273–283 10.1111/j.1748-1716.2006.01552.x 16734764

[CR6] Hashizume T, Takahashi Y, Numata M, Sasaki K, Ueno K, Ohtsuki K, Ishii A (1999) Plasma profiles of growth hormone, prolactin and insulin-like growth factor-I during gestation, lactation and the neonatal period in goats. J Reprod Dev 45(4):273–281

[CR7] Hofseth LJ, Raafat AM, Osuch JR, Pathak DR, Slomski CA, Haslam SZ (1999) Hormone replacement therapy with estrogen or estrogen plus medroxyprogesterone acetate is associated with increased epithelial proliferation in the normal postmenopausal breast. J Clin Endocrinol Metab 84(12):4559–4565 10599719 10.1210/jcem.84.12.6194

[CR8] Jenstad M, Quazi AZ, Zilberter M, Haglerød C, Berghuis P, Saddique N, Goiny M, Buntup D, Davanger S, Haug FMS, Barnes CA, McNaughton BL, Ottersen OP, Storm-Mathisen J, Harkany T, Chaudhry FA (2009) System A transporter SAT2 mediates replenishment of dendritic glutamate pools controlling retrograde signaling by glutamate. Cereb Cortex 19(5):1092–1106 18832333 10.1093/cercor/bhn151

[CR9] Kandiel MM, Watanabe G, Sosa GA, Abou El-Roos ME, Abdel-Ghaffar AE, Li JY, Manabe N, El Azab AS, Taya K (2010) Profiles of circulating steroid hormones, gonadotropins, immunoreactive inhibin and prolactin during pregnancy in goats and immunolocalization of inhibin subunits, steroidogenic enzymes and prolactin in the corpus luteum and placenta. J Reprod Dev 56(2):243–250 20035106 10.1262/jrd.09-159s

[CR10] Kandiel MM, Watanabe G, Abou-El-Roos ME, Abdel-Ghaffar AE, Sosa GA, El-Azab Ael S, Nagaoka K, Li JY, Manabe N, Taya K (2012) Follicular turnover and hormonal association in postpartum goats during early and late lactation. J Reprod Dev 58(1):61–68 21986231 10.1262/jrd.11-012s

[CR11] Knoll-Köhler E, Handke A, Wojnorowicz F (1976) Effect of ovarian sex hormones on the pool of free amino acids in maternal tissues of pregnant rats fed a protein-free diet. J Reprod Fertil 46(1):137–142. 10.1530/jrf.0.0460137 1271334 10.1530/jrf.0.0460137

[CR12] Kohler E, Wojnorowicz F, Borner K (1975) Effects of a protein-free diet on amino acids and sex hormones of rats during the early postimplantation stages of pregnancy. J Reprod Fertil 42(1):9–21. 10.1530/jrf.0.0420009 1110477 10.1530/jrf.0.0420009

[CR13] Li Y, Cao Y, Wang J, Fu S, Cheng J, Ma L, Zhang Q, Guo W, Kan X, Liu J (2020) Kp-10 promotes bovine mammary epithelial cell proliferation by activating GPR54 and its downstream signaling pathways. J Cell Physiol 235(5):4481–4493 31621904 10.1002/jcp.29325

[CR14] López A, Torres N, Ortiz V, Alemán G, Hernández-Pando R, Tovar AR (2006) Characterization and regulation of the gene expression of amino acid transport system A (SNAT2) in rat mammary gland. Am J Physiol Endocrinol Metab 291(5):20 10.1152/ajpendo.00062.2006 16787963

[CR15] Mazzulla M, Hodson N, Lees M, Scaife PJ, Smith K, Atherton PJ, Kumbhare D, Moore DR (2021) LAT1 and SNAT2 protein expression and membrane localization of LAT1 are not acutely altered by dietary amino acids or resistance exercise nor positively associated with leucine or phenylalanine incorporation in human skeletal muscle. Nutrients 13(11):3906 34836160 10.3390/nu13113906 PMC8624011

[CR16] Morotti M, Zois CE, El-Ansari R, Craze ML, Rakha EA, Fan SJ, Valli A, Haider S, Goberdhan DCI, Green AR, Harris AL (2021) Increased expression of glutamine transporter SNAT2/SLC38A2 promotes glutamine dependence and oxidative stress resistance, and is associated with worse prognosis in triple-negative breast cancer. Br J Cancer 124(2):494–505 33028955 10.1038/s41416-020-01113-y PMC7852531

[CR17] Palacín M, Estévez R, Bertran J, Zorzano A (1998) Molecular biology of mammalian plasma membrane amino acid transporters. Physiol Rev 78(4):969–1054 9790568 10.1152/physrev.1998.78.4.969

[CR18] Palii SS, Chen H, Kilberg MS (2004) Transcriptional control of the human sodium-coupled neutral amino acid transporter system A gene by amino acid availability is mediated by an intronic element. J Biol Chem 279(5):3463–3471. 10.1074/jbc.M310483200 14623874 10.1074/jbc.M310483200

[CR19] Pinilla J, Aledo JC, Cwiklinski E, Hyde R, Taylor PM, Hundal HS (2011a) SNAT2 transceptor signalling via mTOR: a role in cell growth and proliferation? Front Biosci 3(4):1289–1299. 10.2741/e332 10.2741/e332 21622135

[CR20] Qi H, Meng C, Jin X, Li X, Li P, Gao X (2018) Methionine promotes milk protein and fat synthesis and cell proliferation via the SNAT2-PI3K signaling pathway in bovine mammary epithelial cells. J Agric Food Chem 66(42):11027–11033 30274521 10.1021/acs.jafc.8b04241

[CR21] Rezaei R, Wu Z, Hou Y, Bazer FW, Wu G (2016) Amino acids and mammary gland development: nutritional implications for milk production and neonatal growth. J Anim Sci Biotechnol 7(20):016–0078 10.1186/s40104-016-0078-8 PMC4818943 27042295

[CR22] Sancak Y, Bar-Peled L, Zoncu R, Markhard AL, Nada S, Sabatini DM (2010) Ragulator-Rag complex targets mTORC1 to the lysosomal surface and is necessary for its activation by amino acids. Cell 141(2):290–303 20381137 10.1016/j.cell.2010.02.024 PMC3024592

[CR23] Telang NT (2022) The divergent effects of ovarian steroid hormones in the MCF-7 model for luminal a breast cancer: mechanistic leads for therapy. Int J Mol Sci 23(9):4800. 10.3390/ijms23094800 35563193 10.3390/ijms23094800 PMC9105252

[CR24] Vaughan OR, Maksym K, Silva E, Barentsen K, Anthony RV, Brown TL, Hillman SL, Spencer R, David AL, Rosario FJ, Powell TL, Jansson T (2021) Placenta-specific Slc38a2/SNAT2 knockdown causes fetal growth restriction in mice. Clin Sci 135(17):2049–2066 10.1042/CS20210575 PMC8410983 34406367

[CR25] Velázquez-Villegas LA, Ortíz V, Ström A, Torres N, Engler DA, Matsunami R, Ordaz-Rosado D, García-Becerra R, López-Barradas AM, Larrea F, Gustafsson J, Tovar AR (2014) Transcriptional regulation of the sodium-coupled neutral amino acid transporter (SNAT2) by 17β-estradiol. Proc Natl Acad Sci U S A 111(31):11443–11448 25056967 10.1073/pnas.1412099111 PMC4128162

[CR26] Velázquez-Villegas LA, López-Barradas AM, Torres N, Hernández-Pando R, León-Contreras JC, Granados O, Ortíz V, Tovar AR (2015) Prolactin and the dietary protein/carbohydrate ratio regulate the expression of SNAT2 amino acid transporter in the mammary gland during lactation. Biochim Biophys Acta 5(64):17 10.1016/j.bbamem.2015.02.011 25701231

[CR27] Yang D, Jiang T, Liu J, Zhang B, Lin P, Chen H, Zhou D, Tang K, Wang A, Jin Y (2018) CREB3 regulatory factor -mTOR-autophagy regulates goat endometrial function during early pregnancy. Biol Reprod 98(5):713–721. 10.1093/biolre/ioy044 29447354 10.1093/biolre/ioy044

[CR28] Yazici E, Ozenc E, Celik HA, Ucar M (2018) Ultrasonographic foetometry and maternal serum progesterone concentrations during pregnancy in Turkish Saanen goats. Anim Reprod Sci 197:93–105 30172605 10.1016/j.anireprosci.2018.08.017

[CR29] Zhu H, Jia Q, Zhang Y, Liu D, Yang D, Han L, Chen J, Ding Y (2022) Regulation of tight junctions by sex hormones in goat mammary epithelial cells. Animals (basel) 12(11):1404. 10.3390/ani12111404 35681868 10.3390/ani12111404 PMC9179430

[CR30] Zwick RK, Rudolph MC, Shook BA, Holtrup B, Roth E, Lei V, Van Keymeulen A, Seewaldt V, Kwei S, Wysolmerski J, Rodeheffer MS, Horsley V (2018) Adipocyte hypertrophy and lipid dynamics underlie mammary gland remodeling after lactation. Nat Commun 9(1):3592. 10.1038/s41467-018-05911-0 30181538 10.1038/s41467-018-05911-0 PMC6123393

