# Supplementary material for: SNAT2-mediated regulation of estrogen and progesterone in the proliferation of goat mammary epithelial cells
Source: Amino Acids. 2024 Feb 23;56:17. doi: 10.1007/s00726-024-03382-w (PMC10891196; doi:10.1007/s00726-024-03382-w)
Supplement: Supplementary file 1 [file 726_2024_3382_MOESM1_ESM.docx]

Supplementary material for

SNAT2 mediated regulation of E_2_ and P_4_ on proliferation of goat mammary epithelial cells

Qianqian Jia^1^., Diqi Yang^1^., Xiaoyue Ma^1^., Yumiao Zheng^1^., Mingxing Ding^1^., Jianguo Chen^1^., Yi Ding., Ming Sun^1^., Hongmei Zhu^1,*^

1College of Veterinary Medicine, Huazhong Agricultural University, Wuhan 430070, China

*** Author for correspondence**

Hongmei Zhu, E-mail: han.dong.1988@163.com

**Materials and Methods**

**Supplementary Figure 1**

**Figure. 1** Plasmid profile of pEGFP-SNAT2-C1 overexpression vector and its multiple cloning site. SLC38A2, SNAT2.

**Supplementary Figure 2**

**Figure. 2** Screening of different SNAT2 interference fragments. (A) The mRNA expression of SNAT2 in GMECs under the treatment of different SNAT2 interference fragments was detected by real-time PCR. (B) The protein expression of SNAT2 in GMECs under the treatment of different SNAT2 interference fragments was detected by western blot analysis. Values are means ± SD from three independent experiments.

***P<0.001, **P<0.01, *P<0.05, *compared with the SiRNA-NC group.

The siRNA sequences are as follows:

SiRNA-NC sequence: sense 5’-UUCUCCGAACGUGUCACGUTT-3’

antisense 5’-ACGUGACACGUUCGGAGAATT-3’

SiRNA-13 sequence: sense 5’-GCUGAGGAAUUUAGGAUAUTT-3’

antisense 5’-AUAUCCUAAAUUCCUCAGCTT-3’

SiRNA-18 sequence: sense 5’-GCUCUGUUCUUCCUGCUAATT-3’

antisense 5’-UUAGCAGGAAGAACAGAGCTT-3’

SiRNA-80 sequence: sense 5’-GCUGGGACAUAAAGCAUUUTT-3’

antisense 5’-AAAUGCUUUAUGUCCCAGCTT-3’

**Supplementary Figure 3**

**Figure. 3** Screening for rapamycin. The cell viability of GMECs treated with different concentration of rapamycin (Rap) was detected by CCK-8 assay. Data were the means ± SD from at least three independent experiments. Values with different superscripted lowercase letter indicate significant difference (P < 0.05).

Figure 2A. The original gel of SNAT2.

Figure 2A. The original gel of β-actin.

Figure 2C. The original gel of SNAT2.

Figure 2C. The original gel of β-actin.

Figure 3B. The original gel of SNAT2.

Figure 3B. The original gel of β-actin.

Figure 4A. The original gel of mTOR.

Figure 4A. The original gel of p-mTOR.

Figure 4A. The original gel of S6K1.

.

Figure 4A. The original gel of p-S6K1.

Figure 4A. The original gel of 4EBP1.

Figure 4A. The original gel of p-4EBP1.

Figure 4A. The original gel of β-actin.
